# Development and international multicentre pilot testing of a postal dosimetry audit methodology for high dose rate brachytherapy

**DOI:** 10.1016/j.phro.2024.100665

**Published:** 2024-11-02

**Authors:** Alexis Dimitriadis, Anna Becker, Krzysztof Chelminski, Pavel Kazantsev, Egor Titovich, Godfrey Azangwe, Liset de la Fuente Rosales, Benjamin Kellogg, Mauro Carrara, Jamema Swamidas

**Affiliations:** International Atomic Energy Agency, Department of Nuclear Sciences and Applications, Division of Human Health, Vienna, Austria

**Keywords:** HDR brachytherapy, Dosimetry audit, Quality Assurance

## Abstract

**Background and Purpose:**

Dosimetry audits are essential for reducing errors in brachytherapy. A postal dosimetry audit methodology was developed and tested in an international multicentre pilot, to assess the accuracy of the Reference Air Kerma Rate of ^192^Ir and ^60^Co brachytherapy sources.

**Materials and Methods:**

A compact phantom made of polymethyl methacrylate was developed to accommodate two catheters, a radiophotoluminescence dosimeter (RPLD) for dose measurements and a Gafchromic (RTQA2) film strip for source position verification. Deviations of the audit setup from TG-43 conditions were quantified experimentally and compared to previous Monte Carlo (MC) simulations. A measurement uncertainty budget was estimated for the RPLD analysis. The methodology was tested in an international pilot study consisting of 59 dosimeter sets among 48 centres from 11 countries.

**Results:**

The experimental correction factors showed good agreement with previous MC simulations, and the total correction factor accounting for non-water equivalence, lack of scatter and beam quality was found to be 1.029 ± 0.009 for ^192^Ir and 1.059 ± 0.007 for ^60^Co sources, to be employed in audit measurement. The total uncertainty budget was estimated to be 2.24 % (k = 1). In the multicentre study, the ratio between measured and reported user dose ranged from 0.968 to 1.049, with all irradiated dosimeter sets within ± 5 %, and 54 out of 59 within ± 3 %.

**Conclusions:**

The methodology was tested in an international multicentre pilot study and has shown good performance validating the uncertainty budget.

## 1 Introduction

Brachytherapy plays a vital role in modern radiation oncology, offering a targeted approach to treat clinical sites, such as cervix. As with any radiotherapy procedure, it is crucial to ensure accurate dose delivery to maximize treatment efficacy and minimize risks. Robust quality assurance, including independent dosimetry audits, is indispensable in maintaining patient safety and treatment quality. Precise determination of absorbed dose is of utmost importance, and therefore, reliable dosimetric systems are required to conduct audits effectively [1], [2].

While audits in External Beam Radiotherapy (EBRT) are well-established [3], [4], [5], [6], audits in brachytherapy are evolving [7], [8], [9], [10], [11], [12], [13], [14], [15], [16]. The characteristics of brachytherapy dosimetry, such as steep dose gradients, high dose fractionation regimes, and inhomogeneous dose distributions within clinical regions, make dose measurements challenging [8]. Published audit studies have mostly focused on measuring source strength with a variety of dosimeters [7], [9], [10], [11], [12], [13], [16]. Other audits have focussed on checking the source position, employing film dosimetry and dedicated phantoms for accommodating clinical applicators [17], [18]. Simple phantom setups with straight catheters are commonly used [7], [10], [11], [19] and geometrical reconstruction tests utilizing specialized phantoms have also been developed [19]. In recent years, efforts were made to conduct more complex end-to-end audits using dedicated phantoms that accommodate clinical applicators, incorporating more steps of the clinical workflow, such as imaging, reconstruction, and planning [9], [15], [16], with advanced audits performing 2D-dose measurements [14].

The postal dosimetry audit programme offered by the International Atomic Energy Agency / World Health Organization (IAEA/WHO) currently provides audits only for EBRT [20], [21], [22]. To complement current services, an effort was initiated to develop a methodology for auditing the Reference Air Kerma Rate (RAKR) of HDR sources with a simple, light weight, compact, cost-effective phantom suitable for remote postal audits with a straightforward, standardised treatment plan [23].

This paper presents the fabrication and characterization of a phantom and dosimetry system, including the calculation of correction factors for the lack of full-scatter, non-water equivalence of the phantom, and quality correction factors acquired through experimental measurements. An uncertainty budget was also developed. The feasibility of the methodology was validated against previous Monte Carlo (MC) simulations [24] and tested in an international multicentre pilot study.

## 2 Materials and methods

### 2.1 Phantom and treatment plan

**Fig. 1:**
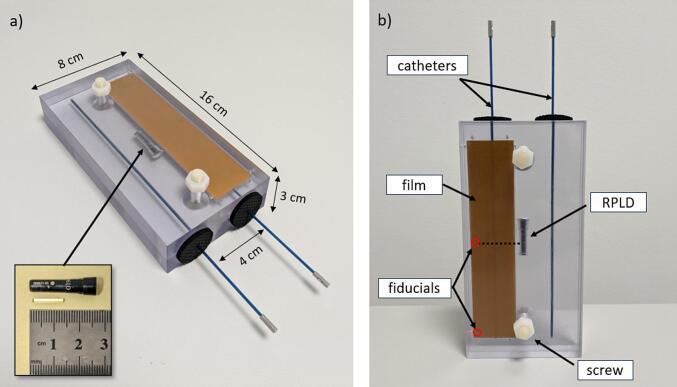
Phantom with inserted plastic catheters and loaded with an RPLD at the centre and a film strip on top of one of the channels, with a) indicated dimensions. The insert in the bottom left corner shows the RPLD capsule and the glass rod with a ruler for scale and b) with indicated components.

Previous MC simulations provided a theoretical basis to manufacture a polymethyl methacrylate (PMMA) phantom design suitable for audits with outer dimensions of 16 cm × 8 cm × 3 cm [24]. This was manufactured out of two 1.5 cm thick slabs, held together by two nylon screws (Fig. 1). In line with the simulated design, the phantom had two parallel channels that were 4 cm apart, allowing the insertion of interstitial plastic catheters that are 1.67 or 2 mm in diameter. A cylindrical Radiophotoluminescent Dosimeter (RPLD), 12 mm long and 1.5 mm in diameter, made of a silver activated phosphate glass (GD-302 M, *Chiyoda Technol Corporation, Japan*) was used for dose measurements. The RPLD was housed inside a watertight high-density polyethylene (HDPE) capsule and positioned in the centre of the phantom, between the two catheters (Fig. 1).

**Fig. 2:**
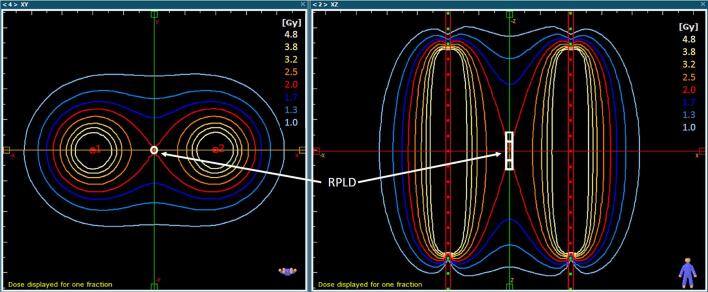
Dose distribution of the reference treatment plan. The 2 Gy isodose line is normalised to a central control point, which coincides with the centre of the RPLD’s sensitive volume indicated by the inner white rectangle. The outer white rectangle represents the full volume of the RPLD.

A simple reference treatment plan was previously designed, that was achievable on all major commercial treatment planning systems (TPS) and deliverable by all major commercial HDR afterloader systems [23]. This consisted of 13 dwell positions in each catheter with uniform dwell times and 5 mm step size. The source dwell times required to deliver 2 Gy to a control point at the centre of the RPLD were calculated using the TG-43 algorithm (Fig. 2) [25]. The final dose calculation, which was used as the reference, was taken from MC simulations [24]. The plan was designed such that the RPLD sensitive volume is in a low dose gradient region (D_min_: 1.99 Gy, D_max_: 2.00 Gy, D_mean_: 2.00 Gy) to ensure that potential source position errors are mitigated, and the dose measured by the RPLD reflects only the accuracy of the RAKR. A Gafchromic film strip (RTQA2 *Ashland Inc., USA*) of 3.2 cm x 14 cm size was placed between the slabs on top of one of the channels, to provide an independent verification that the RPLD was irradiated correctly. A fiducial marker system indicating the centre of the RPLD’s sensitive volume, and the catheter tip was added for position identification during film analysis (Fig. 1b).

### 2.2 Experimental measurement of correction factors

**Fig. 3:**
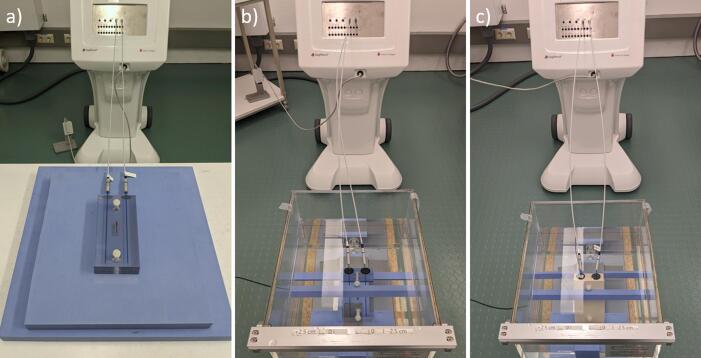
(a) left: PMMA phantom on a table, with 5 cm of solid water slabs in between, connected to the afterloader. (b) centre: PMMA phantom in water, secured by solid water slabs and connected to the afterloader. (c) right: Plastic Water LR phantom in the same setup as shown in (b).

As the proposed audit setup deviates from full-scatter water-equivalent conditions (TG-43) [25], a series of experiments were performed to determine the contributing components. The experiments were conducted with both HDR ^192^Ir and ^60^Co sources (Ir2.A85-2, Co0.A86, SagiNova v.2.2, *BEBIG, Germany*). Plastic catheters of 2 mm diameter (LLA200-KB) were inserted into the phantom and connected to the afterloader with transfer tubes (LAG1000, *BEBIG, Germany*). The same setups simulated in a previous study [24], were investigated experimentally: In setup A, the phantom was placed on a table with sufficient backscatter material underneath (Fig. 3a). In setup B, the phantom was submerged into a water (Fig. 3b). In setup C a phantom of the exact same design, but made of solid plastic water LR (*CIRS, USA*) instead of PMMA was submerged into water (Fig. 3c).

While PMMA is an affordable, transparent, and easily machinable material suitable for large scale postal dosimetry audits, it is not water-equivalent. Plastic Water LR (a non-transparent and relatively expensive plastic material) was selected for setup C as it has been shown to be a near water-equivalent medium for brachytherapy [26], making it suitable for conducting this experiment. Therefore, setup C was considered to represent an RPLD surrounded by water, to obtain the correction factor for non-water equivalence of the PMMA phantom.

By comparing the dose to the RPLD in setup A, *D_a_,* and setup B, *D*_r,p_, a correction factor *k*_s_, accounting for the lack of full-scatter was derived:

(1) $$k_{s}=D_{r,p}/D_{a}$$

Likewise, by comparing the dose in setup B and setup C, *D*_r,w_, a correction factor *k*_m_ accounting for the material perturbation due to the non-water equivalence of the PMMA phantom was derived:

(2) $$k_{m}=D_{r,w}/D_{r,p}$$

For setup A, which is a convenient setup suitable for postal dosimetry audits in hospitals (Fig. 3a), the phantom was placed on a wooden table, on a block of 5 cm of solid water material, as it was previously shown that this is sufficient to ensure that the table material does not significantly influence the dose [24]. For the two setups in water, a plastic tank with dimensions of 30 × 30 × 30 cm^3^ was used to simulate near full-scatter conditions with the respective phantom submerged in the water and secured with solid water supports (Fig. 3b). The source position was verified prior to each irradiation, while phantoms without films were used for convenience.

An RPLD system currently used for EBRT postal audits was utilised for this study, following previously described procedures [22], [27]. Likewise, all irradiations were performed in a three-day window to ensure negligible signal fading corrections [27]. For each setup and source model, 20 RPLDs were irradiated and read on an FDG-1000 reader (*Chiyoda Technol Corporation, Japan)* after preheating and cleaning the detectors. Each RPLD was read out twice by four independent users and using three different readers to improve measurement uncertainty.

The RPLD system was calibrated for absorbed dose measurements with reference to an EBRT ^60^Co beam (X-200, *Nordion, Canada*). To account for the difference in beam quality between this reference beam and the investigated HDR brachytherapy sources, 20 RPLDs were irradiated with 2 Gy in reference conditions positioning the RPLDs at a source-to-axis distance of 100 cm and at a 5 cm depth in a solid water phantom (*Gammex, USA*). By comparing the reference RPLDs (*D*_ref_) to those irradiated with the HDR sources in setup C (*D*_r,w_), a quality correction factor *k*_Q_ accounting for the difference in beam qualities was extracted:

(3) $$k_{Q}=D_{\mathrm{ref}}/D_{r,w}$$

This quality correction factor (*k*_Q_) was applied to transfer the calibration of the RPLD system from ^60^Co beam quality to the respective HDR brachytherapy quality being audited. As a result, the final dose determination for brachytherapy audits was obtained as follows:

(4) $$D=M\times N\times k_{Q}\times k_{s}\times k_{m}$$

where *M* is the RPLD signal corrected for dosimeter sensitivity, fading and readout tray position, *N* is the system’s calibration coefficient in ^60^Co, *k*_Q_, *k*_s_ and *k*_m_ are the correction factors for the beam quality, lack of scatter and perturbation due to non-water equivalence of the PMMA phantom respectively.

The combination of all three correction factors, averaging experimental and MC results where available, was used to calculate the total correction factor to be applied for the brachytherapy audits. The total correction factor *k*_tot_ is therefore given by:

(5) $$k_{tot}=\frac{{(k}_{m}^{exp}+k_{m}^{MC})}{2}\;\times \frac{{(k}_{s}^{exp}+k_{s}^{MC})}{2}\;\times \;k_{Q}$$

### 2.3 Calculation of measurement uncertainty for brachytherapy audits

The measurement uncertainty budget of the RPLD system used in photon and electron beams has been previously reported [22], and most of its components were applied for brachytherapy as well, since they are independent of beam quality. The newly derived brachytherapy-specific uncertainties were thus added to the previous budget. For convenience, one uncertainty budget was employed for both ^60^Co and ^192^Ir sources and the largest uncertainty was budgeted where they differed. The dose calculation uncertainty to the RPLD volume, was simulated with MC methods in a previous work and budgeted here [24]. The brachytherapy-specific uncertainty was added to the previously determined budget [22], [27]. All uncertainty factors were calculated following the BIPM Guide to the Expression of Uncertainty in Measurement [28].

### 2.4 Multicentre pilot study

A pilot study was initiated and 54 centres from 11 different countries were invited to participate and test the developed methodology. While six centres were unable to perform the irradiations, others performed more than one irradiation resulting in a total of 59 dosimeter sets of which 45 were irradiated with an ^192^Ir and 14 with a ^60^Co HDR source, using 49 brachytherapy afterloaders of various models. The auditees received the phantom and a dosimeter set consisting of two films strips and three RPLDs, of which one was a background dosimeter. Compatible and commissioned catheters were provided by the audited centre to ensure compatibility with their transfer tubes and afterloader system. Instructions were provided for the participants to create the required plan (Supplementary Material-1). The auditees were asked to irradiate the two dosimeters in two subsequent deliveries using the audit setup A (Fig. 3a). The dosimeters and films were sent back to the IAEA dosimetry laboratory for analysis and the participating centres were asked for feedback on the audit experience.

**Fig. 4:**
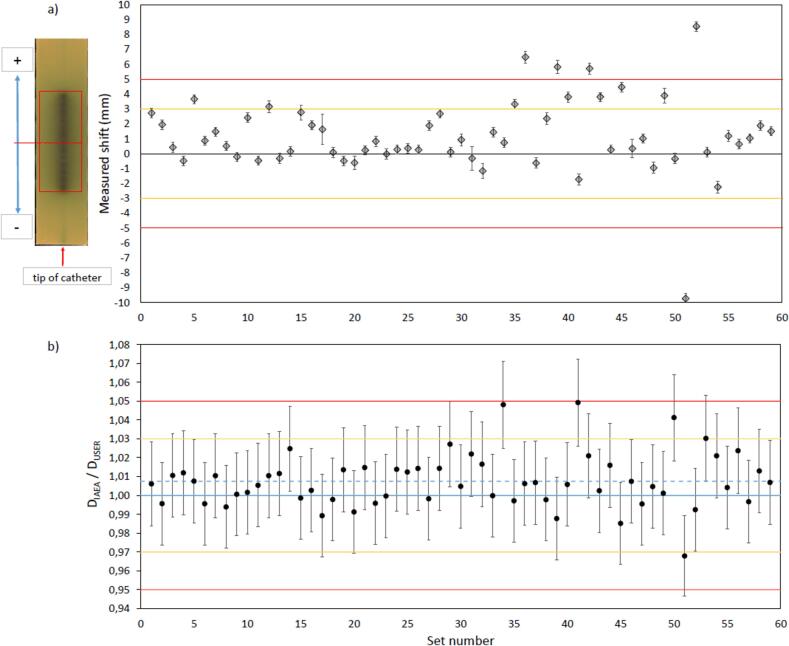
a) The average measured source position shifts for all participating centres (diamonds). the error bars represent the calculated uncertainty based on the two- film measurements for each set, and the uncertainty of the film analysis procedure. the orange lines represent ± 3 mm shifts and the red lines ± 5 mm shifts. b): The corresponding Dose ratio results (D_IAEA_/D_user_) for all participating centres (circles). The orange lines represent ± 3 % dose differences and the red lines ± 5 % dose differences. The average of all sets (1.007) is shown with the blue dashed line. The error bars represent the overall measurement uncertainty of 2.2 % (k = 1). (For interpretation of the references to colour in this figure legend, the reader is referred to the web version of this article.)

The films were scanned on a flat-bed scanner (11000 XL, *Epson, Japan*) in reflection mode at resolution of 300 dpi and analysed using ImageJ (v 1.54f) with an in-house macro following a previously validated methodology [29]. The darkened film area along the source path and its bisection was compared to the fiducial indicating the centre of the RPLD’s sensitive volume, and the difference between the two was defined as the measured source position shift (Fig. 4a). Since two films were irradiated separately, the measured source position was evaluated for each film individually and then averaged. The uncertainty was calculated by combining the uncertainty of the film analysis procedure, ± 0.3 mm [29], and the SD of the two film measurements. The RPLDs were readout following the standard audit procedure, applying all necessary correction factors. The results of the two irradiations were averaged, and the measured dose *D*_IAEA_ was compared to the dose reported by the participants, *D*_user_.

## 3 Results

### 3.1 Correction factors

Experimentally derived correction factors agreed with MC derived correction factors within 0.1 % for ^60^Co and 0.5 % for ^192^Ir and were within the calculated uncertainties (Table 1).

**Table 1:** Experimental results for the correction factors k_m,_ k_s,_ and k_Q_, compared to MC simulated values for k_m_ and k_s_ from a previous study for ^60^Co and ^192^Ir HDR sources (Bebig-Co0.A86 and Bebig-Ir2.A85-2). The total correction factor k_tot_ was calculated as the product of the three correction factors, averaging the experimental and MC results.

		**^60^Co**		^192^**Ir**	
Correction factor		Experiment (current study)	MC simulation [24]	Experiment (current study)	MC simulation* [24]
*k*_m_	Non-water equivalence	1.004 ± 0.003	1.005 ± 0.005	0.993 ± 0.005	0.993 ± 0.009
*k*_s_	Lack of scatter	1.010 ± 0.003	1.009 ± 0.005	1.054 ± 0.004	1.059 ± 0.008
*k*_Q_	Beam quality	1.043 ± 0.006	−-	0.981 ± 0.005	−-
*k*_tot_		1.059 ± 0.007		1.029 ± 0.009	

*MC simulations for the ^192^Ir source were performed using a similar source model (Bebig-GI192M11).

### 3.2 Uncertainty budget

The brachytherapy-specific uncertainties combined with all other contributing components were calculated to have a relative standard uncertainty of 2.24 % for RPLD measurement in postal brachytherapy audits (Table 2).

**Table 2:** Total uncertainty budget for the RPLD system used for brachytherapy dosimetry audits. All components are Type A, unless otherwise stated.

**Component**	**Relative standard uncertainty [%]**
Calibration coefficient	0.69
Calibration coefficient (Type B)	0.54
Reading of a dosimeter	0.15
Individual dosimeter sensitivity correction factor	0.42
Dosimeter positioning during readout	0.18
Dose response non-linearity correction factor	0.83
Fading correction factor	0.01

*Brachytherapy components*	
RAKR (Type B)	1.35
Dose calculation in the RPLD volume (MC derived) [24]	0.90
Total correction factor *k*_tot_	0.83
Combined (k = 1)	2.24

### 3.3 Results of the multicentre study

Fourty-eight out of fifty-four (48/54) centres were able to successfully complete the audit. Out of the 59 irradiated dosimeter sets, 54 showed an averaged shift in the source position within ± 5 mm, and 47 sets were within ± 3 mm, with an average shift of 1.2 mm ± 2.5 mm (Fig. 4a). Out of the 12 sets with a measured shift of more than 3 mm, 11 showed a positive shift, towards the proximal end of the catheter, implying systematic variation in the source position.

The dose ratios (*D*_IAEA_/*D*_user_) ranged from 0.968 to 1.049, with a mean of 1.008 ± 0.014 (SD) (Fig. 4b). When differentiated by radionuclide, the average result for ^192^Ir sets was 1.008 ± 0.015, and 1.007 ± 0.011 for ^60^Co sets. All the measured doses were within ± 5 % of the user-stated dose, and 54 out of the 59 sets were within ± 3 %. Comparing the two individual RPLD doses within one set, the average difference was 1.6 % (± 0.1 %).

## 4 Discussion

A postal dosimetry audit methodology was developed to assess the accuracy of the RAKR of HDR brachytherapy sources. The methodology utilized RPLDs and radiochromic films in a standard plan geometry. The phantom was compact, lightweight, and robust facilitating shipment. The design was cost-effective, vendor-agnostic and did not require any special equipment from the end-users, aside from widely available interstitial plastic catheters, compatible transfer tubes, and solid water blocks. The methodology was tested in participating centres which represent a diverse clinical setting.

A consequence of this audit process was the need for correction factors, as the setup deviated from full-scatter, water-equivalent conditions. Perturbations due to non-water equivalence of the PMMA phantom material and the lack of scatter were quantified in previous MC simulations and were validated experimentally in this work with excellent agreement (within 0.1 % for ^60^Co and 0.5 % for ^192^Ir). The previous MC study [24] investigated the spectral variations within the RPLD volume for different ^192^Ir source models, reporting relatively small mean energy differences of ± 2 keV. In the two setups investigated (Fig. 3a and Fig. 3c) differences up to 15–18 keV within the RPLD volume were reported. It must be noted these MC simulations accounted only for physical dose deposition as the RPLD energy dependence could not be simulated. On the other hand, the RPLD measurements presented in this study were influenced by their innate energy dependence. The excellent agreement between MC-derived and experimentally derived correction factors pointed to a negligible energy dependence of the RPLD within the energy range of 2 keV and confirmed that there is no need for source model-specific correction factors. The MC study has also reported correction factors for the perturbation due to the RPLD density, which could not be verified experimentally. These perturbations were however accounted within the measured *k*_Q_ values. The overall deviation from full-scatter, water-equivalent conditions was therefore assessed through a combination of experiments and MC, which is reflected in the reported uncertainty budget.

The laboratory’s reference system for RPLDs was used to determine quality correction factors cross-calibrating the brachytherapy audit setup for absorbed dose measurements. As this reference system was already implemented, a cross-calibration of the newly developed methodology facilitated the introduction of a new audit service into the existing procedures. An alternative approach that could potentially reduce measurement uncertainty, may involve calibrating an RPLD system directly in brachytherapy qualities. This however may require regular access to a highly reproducible experimental setup in both ^192^Ir and ^60^Co HDR sources for reference irradiations of RPLDs, which may not be a feasible solution for many dosimetry audit providers.

The pilot study showed overall good performance and confirmed the applicability of the calculated uncertainty budget. The results from both the film-based positional verification and RPLD-based dose measurements indicated a distribution typical of a multicentre brachytherapy audit. Most of the irradiated dosimeters were within ± 3 % for dose and ± 3 mm for source position shifts. These results were comparable with other multi-centre dosimetry audit studies using different phantoms and dosimeters [7], [9], [10], [11], [13], [14], [15], [17], [18], and in close agreement to international guidance on uncertainty estimates for HDR brachytherapy [1].

The feedback received from the participants reflected that the procedure was practical to follow and could be completed without any major resource challenges. Some centres highlighted opportunities for more clarity in the instructions (Supplementary Material-1). Some participants performed a CT-scan of the phantom to facilitate reconstruction during the planning process, even though the instructions provided steps to create the plan without using any imaging modalities. This may have introduced some additional errors arising from the reconstruction of the catheters from the imaging during the pilot. Furthermore, six centres were unable to perform the irradiation due to unfamiliarity with the catheters. In addition, at least five of the centres that successfully completed the audit reported limited experience with interstitial catheters in their routine clinical practice and as a result may have also introduced some errors. This was reflected in the outliers of the film measurements, as 11 out of 12 film sets outside the 3 mm tolerance had proximal shifts (towards the after loader). While participants were requested to adjust their treatment plan to account for their specific equipment (Supplementary Material-1), these shifts may be related to mis-calculations for the dead space at the catheter-tip during image-based reconstruction. Although a few studies have reported measured source position shifts through multicentre dosimetry audits [14], [16], [17], [18], different applicators and phantoms were employed. Nevertheless, the reported source position shifts were in good agreement with this study. It is worth noting that despite the observed shifts in this work, the respective dose ratio results were within ± 5 %, due to the deliberate methodological design [23], confirming that any dose deviations were principally related to the RAKR, while limited positional errors during the delivery had no influence on the measurement [29]. Three of the audited centres had relatively large dose deviations of ± 4–5 %. While these were higher than expected, other studies reported similar results [10], [13], [14], [15], which may be partly attributed to measurement uncertainty.

In line with the standard dosimetry audit practices [9], [10], [11], [13], [15], [22], [27], the uncertainty budget employed in this work was limited to dosimetry system components and did not include additional uncertainties from participating centres (ie: TPS calculation algorithm, treatment delivery etc).

To conclude, the correction factors and uncertainty for this postal brachytherapy audit methodology were determined and successfully tested in an international multicentre pilot. This also established a basis for developing a more advanced end-to-end audit incorporating clinical applicators, imaging, reconstruction and dosimetric delivery.

## CRediT authorship contribution statement

**Alexis Dimitriadis:** Conceptualization, Software, Validation, Formal analysis, Investigation, Data curation, Writing – original draft, Writing – review & editing, Visualization, Supervision, Project administration. **Anna Becker:** Software, Validation, Formal analysis, Investigation, Data curation, Writing – original draft, Writing – review & editing, Visualization. **Krzysztof Chelminski:** Conceptualization. **Pavel Kazantsev:** Conceptualization, Methodology, Methodology, Writing – review & editing. **Egor Titovich:** Software. **Godfrey Azangwe:** Methodology. **Liset de la Fuente Rosales:** Writing – review & editing. **Benjamin Kellogg:** Writing – review & editing. **Mauro Carrara:** Methodology, Resources, Writing – review & editing, Supervision, Funding acquisition. **Jamema Swamidas:** Conceptualization, Methodology, Resources, Writing – review & editing, Supervision, Project administration, Funding acquisition.

## Declaration of competing interest

The authors declare that they have no known competing financial interests or personal relationships that could have appeared to influence the work reported in this paper.
