# Supplementary material for: Development and international multicentre pilot testing of a postal dosimetry audit methodology for high dose rate brachytherapy
Source: Phys Imaging Radiat Oncol. 2024 Nov 2;32:100665. doi: 10.1016/j.phro.2024.100665 (PMC11570846; doi:10.1016/j.phro.2024.100665)
Supplement: Supplementary Data 1 [file mmc1.pdf]

IAEA
International Atomic Energy Agency
Atoms for Peace and Development

IAEA Laboratories Seibersdorf

DOSIMETRY LABORATORY, DMRP SECTION, DIVISION OF HUMAN HEALTH

[redacted]

[redacted]

The IAEA DOSE QUALITY AUDIT for BRACHYTHERAPY REFERENCE AIR KERMA RATE

INSTRUCTION SHEET

Please irradiate the dosimeters during the period:

[redacted]

and return them to the address given at the end of the instruction.

GENERAL INSTRUCTIONS

The audit includes the following steps:

- Creating an irradiation plan.
- Performing irradiation of RPLDs with 2 Gy and films according to the plan created.

Please note that on average 2 hours (depending on the source strength) are needed to perform this audit.

The package **with dosimeters supplied to you for irradiation** contains:

- A phantom with a preloaded RPLD capsule and a film strip.
- The 2nd RPLD and film strip for irradiation.
- An RPLD with a blue cap which should NOT be irradiated.
- An envelope for films.

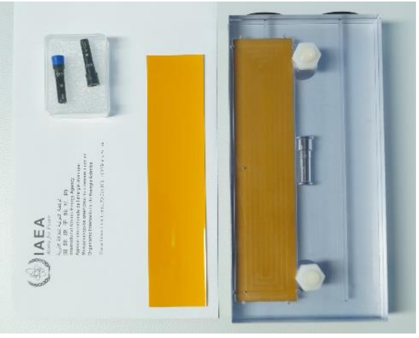

Fig.1. The audit package

SPECIAL NOTE: Please protect the dosimeters and radiochromic films from accidental irradiation, heat (e.g. sunshine), and excessive humidity during storage. Do not store dosimeters in a place where accidental exposure to radiation could occur. Avoid touching the film surface without necessity (e.g. positioning in the holder).

CONFIDENTIALITY: The audit results are kept confidential by the IAEA Dosimetry Laboratory and will not be disseminated without the participant's permission. The overall statistical distribution of the results will be reported.

TECHNICAL INSTRUCTIONS

General remarks

- **Fully familiarize yourself with the audit procedure before attempting to perform the audit.**
- **Handle the phantom, RPLDs and films with care.**
- **Do not leave the box with dosimeters inside the bunker during irradiation.**
- **Do not leave the film to be exposed to direct sunlight or UV radiation.**
- **Do not leave the film or RPLD to be exposed to excessive temperature.**

A. Creating the reference brachytherapy plan

1. Create a new plan
2. Set a prescription dose of 2 Gy in 1 Fraction. Note that you will need to deliver the same plan twice – If your planning system does not allow you to deliver the same plan twice you may need to prescribe 4 Gy in 2 fractions.
3. Import a CT image set if necessary (e.g. some treatment planning systems do not allow to proceed with planning without importing images)
4. Create a control point at coordinate X=0, Y=0, Z=0 (will be used for prescription/normalisation)
5. Insert two Interstitial Applicators that are parallel and 4 cm apart, equidistant from the control point created at step 4 (see Fig. 2).
    - The first applicator starting at X=-2, Y=0, Z=-7 cm and ending at X=-2, Y=0, Z=+7 cm
    - The second applicator starting at X=+2, Y=0, Z=-7 cm and ending at X=+2, Y=0, Z=+7 cm
6. Check the exact coordinates on your TPS screen to ensure that the applicator is as close as possible to the required geometry.
7. If necessary, adjust your offset and indexer length according to your routine clinical procedures for the catheter/transfer tube used.

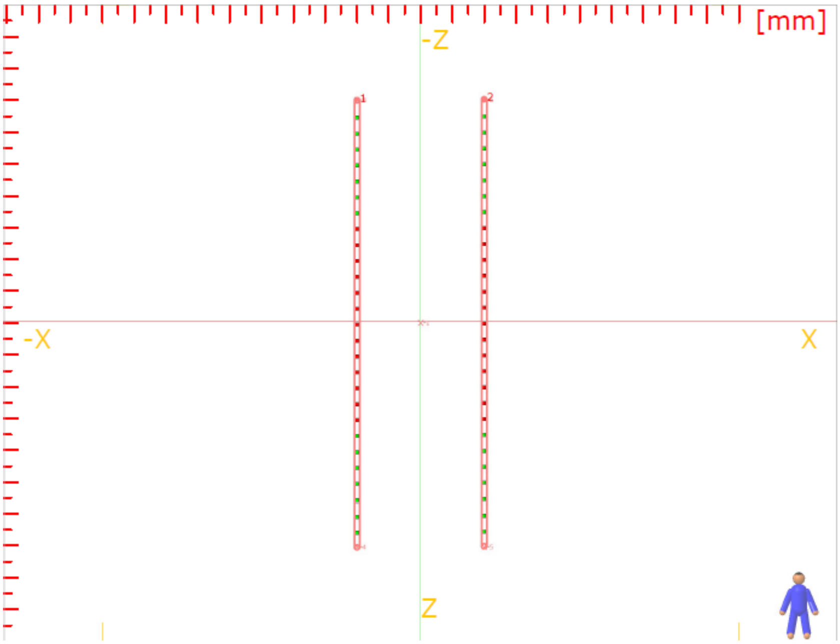

Fig. 2. Positioning of applicators

8. Activate 13 dwell positions in each catheter: two central dwell positions (one in each catheter) at the plane of the central control point (x = 0, y = 0, z = 0), and then 6 additional dwell positions above and 6 additional dwell positions below, with 5 mm spacing (i.e. the first, the central and the last active

dwell shall be at 4, 7 and 10 cm from the catheter tip respectively). Use uniform dwell time (e.g., 10 seconds) for all active dwell positions (Fig. 3).

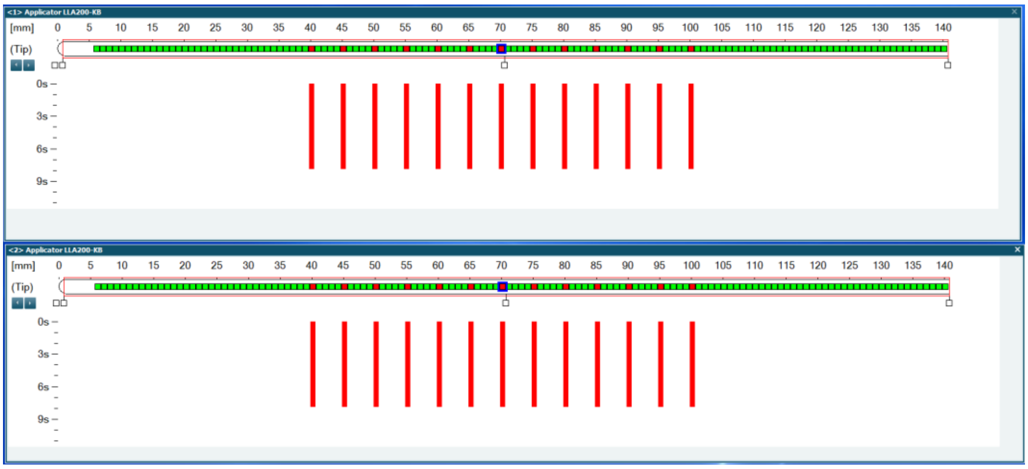

Fig. 3. Uniform dwell times in all 13 dwell positions of both catheters.

9. Using your TG43 dose calculation engine, normalize the plan to deliver 2 Gy to the central control point at X= 0, Y= 0, Z=0. Fig. 4 shows the final geometry, note that the 2 Gy isodose line (Red) is passing through the central control point. Please note that you will have to deliver the plan twice – if necessary prescribe 4 Gy in 2 fractions (2 Gy per fraction).

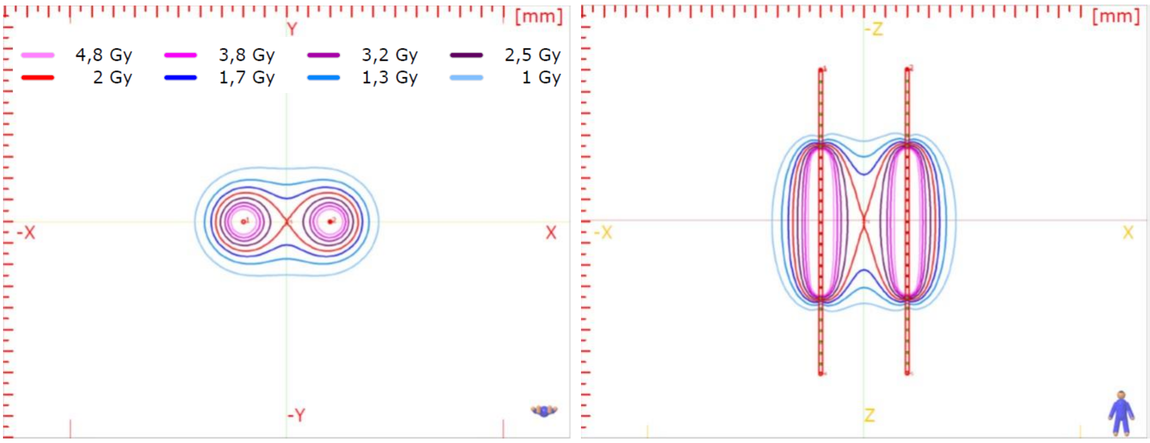

Fig. 4. Final dose distribution. The 2 Gy isodose passes the central point where the RPLD is located.

B. Irradiation of the phantom at the afterloader using the reference plan

1. Transfer the reference plan to the afterloader.
2. Open the plastic transport case with the “IAEA” label on upper side.
3. Take out the phantom and insert your 5 fr or 6 fr plastic catheters in the channels of the phantom.
4. Position the phantom on a solid water slab of at least 5 cm thickness, on top of a table or the patient couch, such that the distances from the room walls to the phantom are at least 1 m. If solid water slab is not available, use water equivalent plastic.
5. Ensure that the phantom is positioned carefully and will not slide during the audit.
6. Connect the transfer tubes to the catheters in the phantom. Ensure that catheters are fully inserted in the phantom. (Fig. 5)

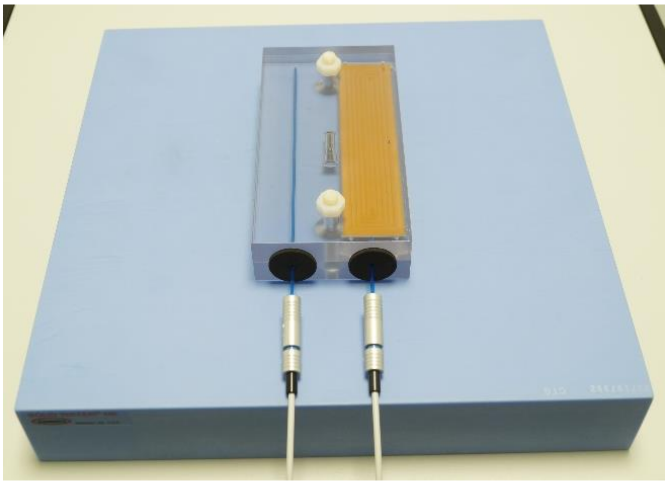

Fig. 5. Irradiation setup.

7. Connect the two transfer tubes to the afterloader as per the reference plan.
8. Perform irradiation using the reference plan.
9. Disconnect the transfer tubes from the catheter needles.
10. Take the phantom in the control area to replace the dosimeters.
11. Remove two plastic screws unlocking the phantom.
12. Lift the upper part of the phantom and remove the irradiated film and RPLD.

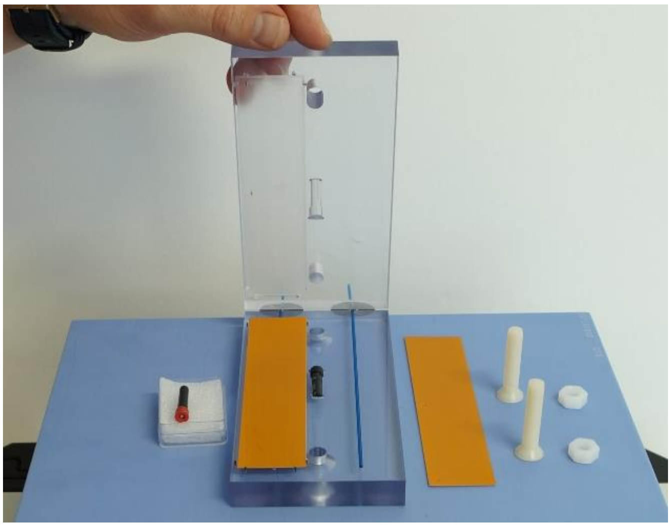

Fig. 6. Changing the dosimeters in the phantom.

13. Mark the irradiated film with the ID number of the RPLD it was irradiated together.
14. Insert the second RPLD and the film into the lower part of the phantom (Fig. 6). Ensure that the film edges are inside the area confined by eight short metal needles at the film corners.
15. Close the phantom and press the upper part of the phantom to allow the fiducials to pierce the film.
16. Lock the phantom with plastic screws and nuts.
17. Reconnect the catheter needles with the transfer tubes
18. Repeat irradiation using the reference plan.
19. Take out dosimeters, mark the film, and put RPLDs in the box and films in the envelope provided.
20. Fill out the datasheet indicating any deviations from the instructions above.

C. Return the audit package

1. Prepare the return package containing:
   - Irradiated films.
   - RPL dosimeters.
   - Phantom (put it into the box with nuts towards the foam insert).

Please send it to the following address:

[redacted]
International Atomic Energy Agency
Dosimetry and Medical Radiation Physics Section
Dosimetry Laboratory
[redacted]

2. Send the filled-out datasheet as an e-mail attachment to [redacted]
